# Lewy bodies, iron, inflammation and neuromelanin: pathological aspects underlying Parkinson’s disease

**DOI:** 10.1007/s00702-023-02630-9

**Published:** 2023-04-16

**Authors:** Peter Riederer, Toshiharu Nagatsu, Moussa B. H. Youdim, Max Wulf, Johannes M. Dijkstra, Jeswinder Sian-Huelsmann

**Affiliations:** 1 Clinic and Policlinic for Psychiatry, Psychosomatics and Psychotherapy, University Hospital Wuerzburg, Margarete-Höppel-Platz 1, 97080 Würzburg, Germany; 2 Department of Psychiatry, University of Southern Denmark Odense, J.B. Winslows Vey 18, 5000 Odense, Denmark; 3 Center for Research Promotion and Support, School of Medicine, Fujita Health University, Toyoake, Aichi 470-1192 Japan; 4 BioShai Ltd., 1 Ha‑Tsmikha St., Yokneam, Israel; 5 Medizinisches Proteom-Center, Medical Faculty, Ruhr-University Bochum, 44801 Bochum, Germany; 6 Medical Proteome Analysis, Center for Protein Diagnostics (PRODI), Ruhr-University Bochum, 44801 Bochum, Germany; 7 Center for Medical Science, Fujita Health University, Toyoake, Aichi Japan; 8 Department of Medical Physiology, University of Nairobi, P.O. Box 30197, Nairobi, 00100 Kenya

**Keywords:** Parkinson’s disease, Iron, Lewy bodies, Inflammation, Neuromelanin, Pathology, α-synuclein

## Abstract

Since the description of some peculiar symptoms by James Parkinson in 1817, attempts have been made to define its cause or at least to enlighten the pathology of “Parkinson’s disease (PD).” The vast majority of PD subtypes and most cases of sporadic PD share Lewy bodies (LBs) as a characteristic pathological hallmark. However, the processes underlying LBs generation and its causal triggers are still unknown. ɑ-Synuclein (ɑ-syn, encoded by the *SNCA* gene) is a major component of LBs, and *SNCA* missense mutations or duplications/triplications are causal for rare hereditary forms of PD. Thus, it is imperative to study ɑ-syn protein and its pathology, including oligomerization, fibril formation, aggregation, and spreading mechanisms. Furthermore, there are synergistic effects in the underlying pathogenic mechanisms of PD, and multiple factors—contributing with different ratios—appear to be causal pathological triggers and progression factors. For example, oxidative stress, reduced antioxidative capacity, mitochondrial dysfunction, and proteasomal disturbances have each been suggested to be causal for ɑ-syn fibril formation and aggregation and to contribute to neuroinflammation and neural cell death. Aging is also a major risk factor for PD. Iron, as well as neuromelanin (NM), show age-dependent increases, and iron is significantly increased in the Parkinsonian substantia nigra (SN). Iron-induced pathological mechanisms include changes of the molecular structure of ɑ-syn. However, more recent PD research demonstrates that (i) LBs are detected not only in dopaminergic neurons and glia but in various neurotransmitter systems, (ii) sympathetic nerve fibres degenerate first, and (iii) at least in “brain-first” cases dopaminergic deficiency is evident before pathology induced by iron and NM. These recent findings support that the ɑ-syn/LBs pathology as well as iron- and NM-induced pathology in “brain-first” cases are important facts of PD pathology and via their interaction potentiate the disease process in the SN. As such, multifactorial toxic processes posted on a personal genetic risk are assumed to be causal for the neurodegenerative processes underlying PD. Differences in ratios of multiple factors and their spatiotemporal development, and the fact that common triggers of PD are hard to identify, imply the existence of several phenotypical subtypes, which is supported by arguments from both the “bottom-up/dual-hit” and “brain-first” models. Therapeutic strategies are necessary to avoid single initiation triggers leading to PD.

## Parkinson’s disease and its major pathological hallmarks

PD is the second most common neurodegenerative disorder after Alzheimer’s disease (Poewe et al. 2017) and is a debilitating neurological disease. It is chronic and categorized by the progressive loss of particular motor functions exhibiting symptoms such as resting tremor, rigidity, bradykinesia, akinesia, gait and posture disorders, risk of falling, problems with fine motoric skills (e.g. writing, facial expression), coupled with non-motor features, including depression, REM-sleep disorder, problems with smell and taste and gastrointestinal disturbances (Obeso et al. 2010; Coelho and Ferreira 2012). These hallmark symptoms develop in stages, as recognized by Braak et al. (2003, 2004). Experimental and neuropathological studies demonstrate that the pathology either is triggered in the gut (Braak et al. 2003) or in the brain (Engelender and Isacson 2017; Foffani and Obeso 2018; Borghammer and Van Den Berge 2019). Alarmingly, several studies have reported an upward trend in the incidence of neurological diseases such as PD in many countries over the past 30 years (GBD Collaborators 2016; Feigin et al. 2019; Ou et al. 2021), thereby highlighting the urgent need to diminish this trend. This presumably could be achieved by understanding the pathogenesis of PD. However, the etiology of the illness remains an enigma. In addition, these elevations in prevalence are further compounded by the aging tendency of the population, because aging is a chief risk factor for PD (Tanner et al. 1996). Interestingly, studies conducted in non-human primates (Collier et al. 2017) have demonstrated an elevation in indicators of midbrain dopamine neuron degeneration with advancing age, including inflammation, mitochondrial dysfunction, oxidative/nitrative destruction, and dysfunctional protein lysosomal system. Furthermore, Collier et al. (2017) reported that these parameters in non-human primates were especially marked in the ventral tier of the SN pars compacta (pc), a region that exhibits a predilection to degeneration in PD in humans (Gibb and Lees 1991; Fearnley and Lees 1991). Extrapolation of these findings suggests that increasing age—accompanied by age-related increases of iron and NM in the SNpc (Zecca et al. 2001)—produces a precondition of the disease and that the illness may manifest in an early PD state if confronted with specific trigger factors (Kouli et al. 2018). These trigger factors may include genetic predisposition, environmental influences (e.g. exposure to metals, smoking, carbon monoxide or pesticides), pathogens (bacteria, viruses) and other unknown culprits (Forsyth et al. 2011; Perez-Lloret et al. 2014; Brenner 2013; Javed et al. 2019; Bekkering et al. 2013; Yamamoto 2013; Reichmann et al. 2022; Ball et al. 2019; Ascherio and Schwarzschild 2016). Still, the precise pathomechanisms underlying the interactions of these factors are unclear. For example, the number of life events was not different between recent onset parkinsonism, PD patients, and healthy controls (Paez-Maggio et al. 2023). Also, there is evidence that smoking may even be protective and/or of beneficial symptomatic efficacy in PD due to nicotinic receptor stimulation (Quik et al. 2009). Despite the advances in discovering many cytotoxic mechanisms and other related factors that may be operating, the precise etiology of the disorder remains intangible. However, it is highly likely to be multifactorial (Pang et al. 2019).

The pathological hallmarks of PD exhibited in the SNpc include neuroinflammation, and the destruction of mainly pigmented dopaminergic neurons coupled with the development and appearance of intra-cytoplasmic inclusions called Lewy bodies (LBs) that are primarily a consequence of aggregates of misfolded α-synuclein (α-syn). These aggregates, alone or in combination, may interfere with the central nervous system’s processing (Kouli et al. 2018).

Table 1 lists several factors believed to promote PD. None of them is always associated with (mild stages of) PD or necessarily leading to PD, supporting the multifactorial concept.

**Table 1:** Factors in the pathology of Parkinson's disease

Factor	Association with PD	Reference examples
Age	Sporadic PD is mostly restricted to the elderly, but only a few percent of the elderly get PD	Moisan et al. (2016)
Gender	PD is ⁓ 1.5 × more prevalent in men than women	Moisan et al. (2016)
Heavy metals/toxins	Subjects with a history of high exposure to manganese and hydrocarbon solvent had a significantly younger onset age (mean age: 50.33 years) than unexposed subjects (mean age: 60.45 years)	Ratner et al. (2014)
	Striatal dopamine activity was lower in three of four individuals exposed to 1-methyl-4-phenyl-1,2,3,6-tetrahydropyridine (MPTP) than in seven controls	Calne et al. (1985)
Lewy bodies (LB)	In 81% of 132 patients diagnosed antemortem with PD α-synucleinopathy was observed in the brain	Dickson (2018)
	About 10% of the unimpaired elderly population display α-S-positive LBs to some extent in the SN	Parkkinen et al. (2005)
Neuromelanin (NM)	NM concentrations in the SN and LC are higher in the elderly	Zecca et al. (2004)
	In PD, NM-positive neurons preferentially die, but: (i) also NM-negative neurons die, (ii) not all neurons with high NM concentration die, and (iii) there is conflicting information as to whether NM concentrations per cell are increased in PD	Mann and Yates (1983)
		Kastner et al. (1992)
		Halliday et al. (2005)
		Carballo-Carbajal et al. (2019)
Iron	Iron concentrations in the SN increase in elderly people	Zecca et al. (2004)
	Iron concentrations in the SN of patients with mild PD are not significantly different from age-matched controls; however, iron concentrations in the SN of patients with severe PD are, on average, much higher than in age-matched controls	Riederer et al. (1989)
		Guan et al. (2017)
Oxidative stress	Glutathione concentrations in the brain, on average, decrease markedly with increasing PD severity, and even in mild PD cases there is a significant decrease compared to controls	Riederer et al. (1989)
	Gluthatione concentrations in melanized nigral neurons on average, but not in all cases, are much lower in PD patients than in controls	Pearce et al. (1997)
Inflammation	In the blood serum of PD patients compared to age-matched controls, interleukin-6 concentrations on average, but not always, are higher	Dobbs et al. (1999)
	In the caudate of PD patients, on average, the concentrations of interleukin-1β and interleukin-6 are much higher than in controls	Mogi et al. (1994a, b)

## The compensatory phase of neurodegeneration in early PD

The destruction of the dopaminergic neurons in the SNpc depletes striatal dopamine. This contributes to the cardinal motor symptoms in PD. A loss of 60% or more of the NM-containing dopaminergic neurons is warranted for the manifestation of the motor dysfunction characteristic of the disease (Bernheimer et al. 1973; Hirsch et al. 1988). However, more recent data suggest a reduction of only 30% of dopaminergic neurons in the SNpc but about a 60% loss of its dendrites (Cheng et al. 2010). Regardless of the precise percentages, the fact that the brain copes and adapts to maintain physiological function despite the progressive loss of dopaminergic cells and dendrites for a long time is a phenomenal demonstration of brain plasticity (Riederer and Wuketich 1976; Mahlknecht et al. 2015; Amoroso et al. 2018). Depleting dopamine levels in the basal ganglia in PD prompts many adaptive alterations at molecular, cellular, and synaptic levels including an increased dopamine turnover—as indicated by the ratios of DOPAC/dopamine and HVA/dopamine (Pifl et al. 2014)—which serve in stabilizing network tasks (Turrigiano 2012). These mechanisms illustrate the operation of homeostatic plasticity in response to a change in the internal basal ganglia environment, particularly in the striatum and the subthalamic nucleus, respectively, the motor loop. Spatiotemporal changes in dopamine, NM, and iron demonstrate that striatal dopaminergic denervation occurs first, followed by abnormal iron metabolism and finally NM changes in the SNpc (Biondetti et al. 2020). In addition, it has been suggested that the progression of the pathology of neurodegeneration adopts a time-linked sequence of anatomic distribution (Alafuzoff and Hartikainen 2017; Luk and Lee 2014). The importance of spatiotemporal developments in PD is demonstrated also in the development of gait disturbances (Doyle et al. 2022).

The loss of dopamine elicits a disbalance of the direct pathway and the indirect pathway in PD (Alexander et al. 1986; Ikemoto et al. 2015; Fieblinger et al. 2014). Therefore, the depletion of dopamine rewires the striatal circuit, increasing the GABAergic inhibitory action and reducing the synapses of the cortico-striatal pathway (Shen and Johnson 2005a, b). Indeed, PET and fMRI studies have endorsed the correlation between depleted synaptic dopamine and the reorganization of the dopamine processes in the brain (Rodriguez-Sabate et al. 2019). As a consequence, upregulation of the postsynaptic dopamine D2 receptors in the striatum was reported to be the highest in the contralateral side to the predominant motor features in unmedicated early PD (Kaasinen et al. 2000, 2021). Interestingly, studies using PET and single-photon emission computed tomography have shown that the increase in striatal D2 receptors is selective to PD and not observed in other basal ganglia disorders that share a marked loss of dopamine, such as multiple systems atrophy and progressive supranuclear palsy (Kaasinen et al. 2021).

Subsequently, after an average of 4.4 years after the onset of motor symptoms, this D2 upregulation is reversed and is directly related to treatment with dopaminergic-related drugs (Kaasinen et al. 2021; Thobois et al. 2004). In contrast, D1-receptors densities are not changed compared to controls (Cropley et al. 2008; Shinotoh et al. 1993; Rinne et al. 1991).

## Lewy bodies and their staged appearance in PD

LBs are inclusion bodies—aggregates—of which the major component is ɑ-syn protein oligomerized into fibril formations. The toxic potential of LBs is demonstrated by experimental studies showing that LB extracts from PD brains obtained post mortem trigger ɑ-syn pathology and neurodegeneration in mice and monkeys, which naturally do not show symptomology of PD (Recasens et al. 2014). According to Braak et al. (2003, 2004), in the PD pathology stages 1 and 2, which are pre-symptomatic, LBs are generated in the gut and spread via the vagus nerve/dorsal motor nucleus of the vagus to the brain. From stages 3 and 4, such observations are also made for the locus coeruleus (LC), the raphe nuclei and the SNpc, and in stages 5 and 6, cortical and limbic areas additionally get involved. Thus, according to this hypothesis, PD neuropathology does not start in the SNpc itself, despite that SNpc pathology is the dominant immediate cause of many PD symptoms.

The vagus nerve is the major direct route for the “gut-brain-axis” through which the gut and brain communicate and affect each other (Menozzi et al. 2021). Because the first PD symptoms usually arise in the dorsal motor nucleus of the vagus, and because in PD patients LBs were found in the Auerbach's and Meissner's plexuses in the enteric nervous system (Wakabayashi et al. 1988), it was hypothesized that PD is initiated by stressors from the enteric system (Braak et al. 2004; Przuntek et al. 2004; Forsyth et al. 2011). It has been suggested that peripheral immune cells such as T lymphocytes infiltrate the brain, gather in the SN, and release pro-inflammatory cytokines that induce dopaminergic cell death (MacMahon Copas et al. 2021). Indeed, gastrointestinal disturbance and especially constipation are a significant non-motor complaint in many patients with PD (Berg et al. 2015; Camerucci et al. 2022). Gastrointestinal disturbance is suggested for the initiation of a PD subgroup, whereby inflammatory agents [such as lipopolysaccharides (LPS)] produced by some gut pathogen may be a trigger that induces misfolding of α-syn and its resulting aggregation in the enteric nervous system. Subsequently, these inclusions may be transported through the gut–brain axis via the vagus nerve to the dorsal motor nucleus of the vagus and the brain regions, either through direct microbe mediation or indirectly via microbe interaction with enteroendocrine cells and gesturing afferent neurons (Bellono et al. 2017) (Table 2).

**Table 2:** Hypotheses underlying the onset of Parkinson’s disease

Model	Characteristics	Reference examples
Brain-first	Pathology is triggered in the brain, several mechanisms are under debate:	
	Cortico-striatal excitation stress causing retrograde striato-nigral degeneration	Foffani and Obeso (2018) and Cheng et al. (2010)
	“Threshold-hypothesis”, suggesting various factors adding up to cause pathology in the brain	Engelender and Isacson (2017)
	Pathology may show laterality	Riederer et al. (2018)
Bottom-up/body-first	Pathology is triggered in the gut	Braak et al. (2003), Przuntek et al. (2004) and Forsyth et al. (2011)
	Spreads via vagus nerve/dorsal motor nucleus of the vagus to the brain	
	Symmetrical brain pathology	Borghammer and Van Den Berge (2019)
Dual-hit	PD pathology may be triggered through the olfactory system and the motor nucleus of the vagus at the same time	Braak et al. (2003), Braak et al. (2006) (https://doi.org/10.1007/s00441-004-0956-9) and Hawkes et al. (2007) (https://doi.org/10.1111/j.1365-2990.2007.00874.x)

Animal studies also support the transportation role played by the vagus nerve, as demonstrated by the absence of spread of α-syn fibrils from the gut to the brain after the administration of α-syn fibrils into the gut muscularis of mice that had undergone truncal vagotomy (Kim et al. 2019). The “communication” between resident gut flora and the brain, also called the microbiome–gut–brain axis, is vital for its neural development and functioning (Klann et al. 2022). This has been demonstrated by Pan-Montojo et al. (2010) showing that the mitochondrial toxin rotenone injected into the gastro-intestinal tract of mice induced ɑ-syn dependent pathology and spread to the brain. Vagotomy completely interrupted this ɑ-syn spreading.

It has been suggested that a “leaky” intestinal epithelial barrier of the colon in PD patients may allow pathogenic components from the gut lumen or their induced pro-inflammatory cytokines to directly stimulate ɑ-syn aggregation in the enteric nervous system (Van Ijzendoorn and Derkinderen 2019).

However, recent evidences indicate several possible routes of PD pathology. Besides (I) the “bottom-up hypothesis” (the gut–brain axis hypothesis) created by Braak et al. (2003), (II) Braak realized that pathology could be triggered also via the olfactory system. He called this the “dual-hit-hypothesis” (Braak et al. 2003). The bottom-up-hypothesis has been challenged soon after its publication and the major argument was that only for about 50% of PD patients neuropathological and clinical examinations accounted for this hypothesis (Parkkinen et al. 2008; Attems and Jellinger 2008). Criticism on Braak’s bottom-up-hypothesis, as summarized by Rietdijk et al. (2017), is several-fold: (1) Braak and coworkers developed this hypothesis only by detecting LBs in the gut, the dorsal motor nucleus of the vagus as well as a number of brain regions without proving a link between the observations; (2) these authors did not correlate their findings to clinical protocols; (3) they did not follow the cases that did not show LBs in the vagus nerve but did show LBs in various brain areas (Attems and Jellinger 2008; Parkkinen et al. 2008; Kalaitzakis et al. 2008; Jellinger 2003, 2019a; Zaccai et al. 2008; Beach et al. 2009); (4) there is no correlation between the severity of LBs pathology in the dorsal motor nucleus of the vagus and in limbic and neocortical brain areas (Kingsburg et al. 2010), while LBs pathology in the dorsal motor nucleus of the vagus does not correlate to olfactory dysfunctions and 27–33% of PD patients did not show LBs pathology in the peripheral nervous system (Lebouvier et al. 2011); (5) LBs pathology has been detected in the olfactory system only in the early phase of PD (Beach et al. 2009), thus not supporting the dual-hit-hypothesis; (6) a number of studies point to the view that LBs pathology occurs in the brain, including the SN but not in the dorsal motor nucleus of the vagus (Jellinger 2003; Parkkinen et al. 2003; Orimo et al. 2007); (7) more and more evidences suggest that the cardiac (NM-free) sympathetic nerves are already affected in the early stages of PD (Orimo et al. 2007; Borghammer and Van Den Berge 2019), findings which have not been addressed by Braak et al. (2003). The review by Rietdijk et al. (2017) reflects all this in detail and is an elegant study exploring additional hypotheses, which nowadays concentrate on a “brain-first” model of LBs pathology (Borghammer and Van Den Berge 2019) (Table 2).

Indeed, several hypotheses (III) have been put forward to explain these discrepant views. (IIIa) Foffani and Obeso (2018) postulate a cortico-striatal excitation stress to be responsible for a retrograde striato-nigral degeneration. Support for this is given by data from Cheng et al. (2010), showing that in early PD only 30% of nigral dopaminergic neurons are degenerated but about 60% of its dendrites. (IIIb) Another causal pathology is the “threshold-hypothesis” put forward by Engelender and Isacson (2017) favouring a variety of vulnerability factors, of which the sum trigger PD pathology starting in the brain. (IIIc) Borghammer and Van Den Berge (2019) claim a PNS-first subtype and a CNS-first subtype of PD. While the former is characterized by a marked autonomic damage before the involvement of the dopaminergic system, the latter is characterized by nigro-striatal dopaminergic dysfunction prior to involvement of the autonomic PNS (Borghammer and Van Den Berge 2019); imaging studies of REM-sleep disorder positive and negative PD patients as well as histological studies support this.

In summary, there is recent evidence to support the idea that various routes of pathology exist, which may be causal for various subtypes of PD. However, the variety of hypotheses are also indicative for (1) an insufficiency of experimental as well as human post-mortem studies, (2) a lack of knowledge regarding the PD patients’ genetic and environmental background and (3) a lack of knowledge regarding the interactions of the patients metabolic, immunological and genetic factors that may correspond to a vulnerability level causal for PD.

## Familial PD mutations agree well with observations of protein aggregation and oxidative stress in sporadic PD

For helping to understand the causes of sporadic PD, those of familial PD should be studied. Familial PD is characterized by genetic mutations that increase the chance of PD, and can also affect young people. There are about 20 different of these mutations known, and although they cause different kinds of cellular stress some general biochemical principles are recognizable.

The mechanism(s) underlying the appearance of the α-syn inclusions could be related to an overexpression due to some genetic defect or/and a malfunction in its protein breakdown system and misfolding and accumulation of α-syn may be linked to some defect in its gene, *SNCA*. In 1997, the first pathogenic *SNCA* mutation was reported in PD families (Polymeropoulos et al. 1997). Subsequently, the same mutation was found in familial PD patients mainly of Greek descent (Athanassiadou et al. 1999). Over the years that followed, several other mutations in the *SNCA* gene have been identified, including A53V, A53E, A30P, A30G, E46K, G51D and H50Q (Zarranz et al. 2004; Lesage et al. 2013; Pasanen et al. 2014; Liu et al. 2021). Moreover, mutations in *SNCA* and other PARK genes (including *LRRK2* and *VPS35*) have been linked to autosomal dominant PD (Chelban et al. 2018). It could be undeniably concluded that point mutations in the *SNCA* gene promote early-onset PD characterized by a rapid progression and marked cognitive deterioration (Li et al. 2001; Gialluisi et al. 2021).

It has been suggested that overexpression of *SNCA/*α-syn may impact microRNA expression (Recasens et al. 2016). This may induce the neuro-destruction cycle characteristic of PD since microRNA can influence the accumulation of toxic proteins, which can affect neuronal survival (Eacker et al. 2009). However, the notion is important that only some of the carriers of PD susceptibility alleles do get the disease (Pankratz and Foroud 2007; Langmyhr et al. 2021).

Alternatively, some dysfunction in α-syn degradative proteolytic mechanisms may ascribe to protein accumulation. It has been suggested that the mutated protein serves a role in autophagy and the destruction of neurons. Thus, it is conceivable that genetic defects in the *SNCA* gene may ascribe to misfolding, and in addition, the abnormal protein may impede its breakdown by affecting destructive autophagic mechanisms. Indeed, oxidation of the methionine component of aggregated α-syn by agents such as hydrogen peroxide leads to the inhibition of its proteasomal destruction (Alvarez-Castelao et al. 2014) and lysosomal dysfunction (Ihara et al. 2012; Koh et al. 2019).

Among the mutations that cause increased risks for PD development, some directly promote abnormal protein aggregation, such as for example mutations or copy number increases in the ɑ-syn gene *SNCA* and mutations in the ubiquitin ligase Parkin gene *PARK2* (Kitada et al. 1998); other mutations directly enhance oxidative stress or interfere with the proper functioning of mitochondria, such as for example mutations in the antioxidant DJ-1 gene *PARK7* (Chen et al. 2022; Di Nottia et al. 2017; McCoy and Cookson 2011) and the kinase PINK1 gene *PINK1* (Barodia et al. 2017; Ge et al. 2020; Gautier et al. 2008). This agrees well with observations for sporadic PD, as it was found to be associated with mitochondrial deficits (Mizuno et al. 1989; Reichmann and Riederer 1989; Shapira et al. 1989). The reasons for a loss of respiratory chain activity is not known but might be due to endogenous neurotoxins, like aldehydes or environmental toxins like MPTP (1-Methyl-4-phenyl-1,2,3,6-tetrahydropyridin; Langston 2017), TaClo (1-trichloromethyl-1,2,3,4-tetrahydro-beta-carboline; Sontag et al. 1995; Riederer et al. 2002), pesticides etc., overexpression of ɑ-syn (Riederer et al. 2019 for review), iron accumulation in the SN leading to oxidative stress (Riederer et al. 2021 for review), and loss of antioxidative capacity by loss of antioxidative molecules such as glutathione or even heat shock proteins (Riederer et al. 1985; Sian et al. 1994; Dexter et al. 1994; Gruenblatt et al. 2004; Mena et al. 2015). In addition, the transcriptional co-activator PGC-1α has been found to control the mitochondrial function of nigral neurons accumulating α-syn, which may be critical for gender-dependent vulnerability to PD (Cirion et al. 2015). Furthermore, several types of age-, disease-, or lifestyle-related toxins can stimulate these processes (Forsyth et al. 2011; Perez-Lloret et al. 2014; Brenner et al. 2013; Javed et al. 2019; Bekkering et al. 2013; Yamamoto et al. 2013; Reichmann et al. 2022; Ball et al. 2019). Moreover, oxidative stress—through oxidative deamination—leads to an increase in free aldehydes, which in combination with a PD-characteristic decrease in aldehyde dehydrogenase 1A1 leads to free aldehydes changing the structure of ɑ-syn and thereby contributing to LBs formation (Molochnikow et al. 2012; Michel et al. 2014). Indeed, DOPAL has been found to be even more toxic than dopamine in experimental designs (Lamensdorf et al. 2000; Goldstein 2021). Often considered the most critical for PD development, increased levels of free iron, derived from outside the cell and increasing in the brain with age (Markesbery et al. 1984), enhance the production of reactive oxygen species (ROS). These ROS in turn then can change ɑ-syn structure (Xiao et al. 2018; Li et al. 2001; Ruf et al. 2019).

In summary, both familial and sporadic PD involve oxidative stress and/or protein aggregation as the likely causes, and these two pathological processes promote each other. The third player that can join this pathological cascade is inflammation, with a role for glial cells (Bernaus et al. 2020; Kumar et al. 2012; Hashioka et al. 2021). Although the different possible PD causes do promote each other, their relative contributions differ per case and can help explain the large differences in PD pathologies and symptoms from patient to patient. For example, in some types of familial and non-familial PD, LBs are not commonly detected (Schneider and Alcalay 2017; Marras et al. 2016; Pont-Sunyer et al. 2017; Jellinger 2019b). From a broader viewpoint, it should be realized that there is overlap in the stressors and pathologies of different neurodegenerative diseases (e.g. PD, Alzheimer’s disease, and Lewy body dementia) that prohibit their simplistic interpretation as pure synucleinopathies, tauopathies, or amyloidopathies, and these different types of protein aggregation are often found in the same patient (Jellinger 2012).

## A closer look at ɑ-synuclein and LBs

ɑ-Syn has an important function for synaptic vesicles and for synaptic processes (Spillantini et al. 1997). For example, it interacts with synapsin (Bieri et al. 2018) and transports proteins and lipids (Oliveira et al. 2021; Rebelo et al. 2021; Taoufik et al. 2018; Verma et al. 2022). However, it has a propensity for forming aggregates, for example under conditions of increased iron concentration or lipid peroxidation (Xiao et al. 2018; Li et al. 2011). LBs contain ɑ-syn as their major component, but in addition contain at least 70 different components including metals, various (phospho-)proteins, and (phospho)-lipids. Many of the proteins in LBs are highly ubiquinated (Wakabayashi et al. 2007; Beyer et al. 2009).

ɑ-Syn is 140 amino acids long and has four tyrosine residues. Tyrosines can easily be oxidized and efficient peroxidative oxidation of ɑ-syn requires its tyrosines (Olteanu and Pielak 2004; Ulrih et al. 2008; Ruf et al. 2019). In particular, the three tyrosines in the C-terminal of ɑ-syn were found critical for its propensity for aggregation/fibrillation (Ulrih et al. 2008). As for tyrosine oxidation in regard to ɑ-syn and PD, it is also relevant that the oxidation of free tyrosine produces L-DOPA. Indeed, DOPAnization of tyrosine in α-syn by tyrosine hydroxylase (TH) leads to the formation of oligomers (Jin et al. 2022). L-DOPA has pro-oxidant properties and can modify ɑ-syn (Post et al. 2018) and so increase the sensitivity to oxidative stress and degeneration of catecholaminergic neurons (Lipski et al. 2011). Interestingly, ɑ-syn also has a ferrireductase capacity that promotes the conversion of Fe^3+^ into Fe^2+^ and thereby ROS production, and so may additionally contribute to oxidative stress, α-syn fibrillation and neurodegeneration (Olivares et al. 2009; Rogers et al. 2011; Davies et al. 2011; McDowal and Brown 2016; Sian-Huelsmann and Riederer 2021).

Tyrosines are not the only amino acids of which the oxidation may contribute to ɑ-syn cytotoxicity. Namely, the N terminus of ɑ-syn is essential for its efficient degradation by the proteasome, but oxidation of the methionines at ɑ-syn positions 1 and 5 as observed in LB can reduce this degradation (Alvarez-Castelao et al. 2014).ɑ-syn transfer occurs in two different steps: the release of pathogenic ɑ-syn species from infected cells and their uptake into healthy cells via passive or active endocytic pathways in a stereotypical spatiotemporal spreading across different tissues and brain areas (Neupane et al. 2022). The presence of misfolded/altered α-syn structures in the neurons and glial cells of the brain is not specific for PD and common to a group of neurodegenerative disorders called α-synucleinopathies. This group includes PD, PD with dementia, Lewy body disease dementia, multiple systems atrophy, and rare neuronal axonal dystrophies (Kaufmann and Goldstein 2010; McCann et al. 2014). Although the precise pathways have not been clarified, it appears that α-syn is a key player in the intricate labyrinth of the pathogenesis of PD. However, this inclusion is not specific to PD only. In addition, PD occurs in some instances in the absence of LBs, for instance, PD without nigral degeneration-SWEDD (Ling et al. 2016; Milber et al. 2012), post-encephalitic PD (Jellinger 2009), acute MPTP-induced parkinsonism (Burns et al. 1984), PINK 1 autosomal early PD (Takanashi et al. 2016; Schneider and Alcalay 2017) and LRRK2 (PARK 8) late-onset PD (Pont-Sunyer et al. 2017).

It is unclear whether α-syn/LBs formation is a primary or a secondary event to oxidative stress since both features—depletion of reduced glutathione (Dexter et al. 1994) and the presence of LBs (Gibb and Lees 1988)—are present in the SN of early asymptomatic PD and incidental Lewy body disease. As α-syn has the propensity to exacerbate the disturbing cellular redox equilibrium by its ability to function as the enzyme ferrireductase (Sian-Hulsmann and Riederer 2020, 2021) it can therefore contribute to the ironII/III dyshomeostasis observed in the symptomatic phase of PD (Sofic et al. 1988; Dexter et al. 1989).

Despite the plethora of evidence relating the aggregated α-syn inclusions/LBs to the pathomechanisms in PD, it is unclear whether it is a cause or consequence of the disease. Multiple studies have proposed that LB production marks a protective role; however, if aberrantly folded α-syn is enclosed in LB structures it may contain its harmful, detrimental cellular effects (Olanow 2004). In contrast, others using biochemical, integrative omics, and imaging, have hypothesized that the formation of LBs is not merely an α-syn fibril production but rather a principal factor related to α-syn neurotoxicity (Mahul-Mellier et al. 2020). The cellular havoc produced by the toxic form includes mitochondrial dysfunction and cellular and synaptic disturbances (Sian-Hulsmann et al. 2015).

The question arises whether there is a link between LBs pathology, neuronal loss, and PD symptoms (Rietdijk et al. 2017). While there are strong evidences put forward by genetic studies that LBs pathology is of major importance for PD pathology, Rietdijk et al. (2017) summarize critical aspects which are commonly neglected like: (1) diagnosis of motor symptoms or dementia correlates to wide-spread brain LBs pathology only in about 34% of PD patients (Parkkinen et al. 2008); (2) only about 10% people with LB pathology in the SN, dorsal motor nucleus of the vagus and/or basal forebrain are diagnosed with PD (Parkkinen et al. 2005); (3) neurodegeneration in the SN might precede LB pathology (Milber et al. 2012). Unfortunately, Braak et al. (2003) lack both clinical documentation and information about the neuronal cell loss especially in the SN. However, the latter correlates with motor symptoms (Greffard et al. 2006). Moreover, LBs pathology (Beach et al. 2009) is not correlated to dopaminergic cell loss in the striatum and it has been questioned whether it may be correlated to that of the SN (Beach et al. 2009; Parkkinen et al. 2011). Experimental studies using long-term application of MPTP in mice show accumulation of LB-like aggregates—in contrast to when using acute MPTP application—and this seems to indicate that LBs pathology is secondary to a metabolic process triggering PD (Fornai et al. 2005; Meredith and Rademacher 2011). Of interest, streptozotocin, which dysregulates the insulin/insulin receptor pathway, leads to amyloid-like aggregates in rats only after about 6 months post-application and thus mimics pathology associated with Alzheimer disease. Therefore, also in this model, pathological aggregates are only late signs of pathology (Knezovic et al. 2015).

The reviews on these topics by Rietdijk et al. (2017), Visanji et al. (2013), Riederer et al. (2019, 2021), Jellinger (2019b), and Urban et al. (2020) point to the conclusion, that LBs pathology is an important factor but at least in sporadic PD is rather a disease progression as well as pathological multiplication factor. In addition, the “brain-first-hypotheses” are able to explain the laterality (Riederer et al. 2018) of early PD pathology as the pathology may start predominantly in one hemisphere, while the bottom-up-hypothesis rather suggests a symmetrical brain pathology (Borghammer and Van Den Berge 2019).

## The role of iron

Iron performs critical functions in the human brain for processes such as oxygen transport, oxidative phosphorylation, myelin synthesis, production and metabolism of neurotransmitters, nitric oxide metabolism, neuromelanin storage in the SN and LC, and ferritin binding in oligodendrocytes and microglia (for a review see Riederer et al. 2021). However, in PD SNpc, large increases in iron are found compared to age-matched healthy controls (Sofic et al. 1988; Dexter et al. 1987; Hirsch et al. 1991; Faucheux et al. 2003; Foley et al. 2022), and within dopaminergic neurons this contributes to oxidative stress and cell death (Youdim et al. 1989; Zecca et al. 2004; Hare and Double 2016; Mochizuki et al. 2020; Riederer et al. 2021; Foley et al. 2022). The increases of especially ferric iron (Fe^3+^) in PD SN are more or less linearly correlated with the decreases in dopamine production. The reasons for the increased iron levels in PD brain may, for example, be an increase in iron uptake through the blood–brain barrier (Oestreicher et al. 1994) and/or a decreased release from cells because of deficiencies in the transmembrane iron transporter molecule ferroportin (Foley et al. 2022). The toxicity of iron was proven in rats, as the intranigral injection of iron induced a reduction in dopaminergic activity, and this effect could be attenuated by treatment with an antioxidant (Ben-Shachar and Youdim 1991; Wesemann et al. 1994). Furthermore, experimental animal studies showed, that reduced cerebral blood flow (cerebral oligemia) flow PLUS enhanced iron concentration within striatal tissue potentiated cognitive deficits and reduced reaction times (Sontag et al. 2006), suggesting, that normal cerebral blood flow is a requirement for reducing the risk for PD. In this regard, the notion is of interest that weak but significant iron peaks similar to those of a synthetic—iron (3) complex were seen only in the intraneuronal highly electron-dense granules of SNpc cells of PD brains with the highest levels in a case of PDD (Jellinger et al. 1992).

The antioxidant enzyme superoxide dismutase converts superoxide radicals into hydrogen peroxide, which can cause grave cellular harm in elevated nigral iron levels in PD (Sofic et al. 1988) via catalyzing the Fenton reaction to produce highly reactive and toxic hydroxyl radicals. The imbalance between the production of reactive free radicals and the depletion of antioxidant defenses such as nigral glutathione (Sofic et al. 1992; Sian et al. 1994) contributes to these destructive cellular processes (Dias et al. 2013) in PD. Indeed, many in vitro studies suggest that reactive oxygen species released by oxidative stress can diminish brain plasticity and synaptic signaling (O’Dell et al. 1991; Salim 2016). Thus, oxidative stress contributes to the accumulation of the misfolded α-syn by impeding its breakdown and the extent of LBs pathology. Furthermore, it has been implicated as a major contributor to the neurodegeneration in PD (Götz et al. 1990; Jenner 2003) or, for that matter, other α-synucleinopathies (Giasson 2000). Iron as an important pathological player is demonstrated by experimental work showing that pathology induced by MPTP or 6-OHDA is antagonized by iron-chelators (Ben-Shachar et al. 1991a, b; Shachar et al. 2004; Wesemann et al. 1995; Youdim et al. 2004a, b, 2005; Grünblatt et al. 2000, 2006; Mandel et al. 2004, 2006; Haskova et al. 2022).

In summary, iron-induced pathology is an important fact in the cascade of toxicity underlying PD. This is substantiated by the fact, that (1) iron injected into the SN of rats affect the dopaminergic nigro-striatal system similar as in PD and (2) iron-induced oxidative stress contributes to destructive cellular processes as well as to accumulation of misfolded α-syn, thus contributing to the progression of the disease.

## The role of neuromelanin

In the SNpc of PD patients, Hirsch et al. (1988) found a 77% reduction compared to healthy controls in the number of catecholaminergic tyrosine hydroxylase-positive neurons. The reduction was higher in NM^+^ neurons than in NM^−^ neurons, and the authors concluded an increased vulnerability associated with NM production at both the tissue and cellular level. Other than the high amount of NM, another possible reason for the vulnerability of the SNpc might be its high cell density (Ma et al. 1995; Ross et al. 2004), which as such could promote the spreading of pathologies. In PD SNpc NM^+^ neurons, frequently, LBs are found in close proximity to both NM granules and the neural membrane (Sian-Hulsmann et al. 2015). Ultimately, the LBs appear to destroy the neurons by breaking them open. LBs are largely proteinaceous and can easily be metabolized from the extracellular environment. In contrast, the released NM complexes are more inert and some observations suggest that they may be transported out of the brain by the immune system and could even end up in the spleen (E. Csanda, personal communication). As such, the notion is of interest, that melaninomacrophages (phagocytic cells) have been detected in ectothermic vertebrates in spleen, liver and kidney cells as reviewed by Dubey and Roulin (2014; see also Michalczyk et al. 2009). From these vague evidences, we propose that the metabolism of NM requires further experimental studies.

NM efficiently binds and inactivates iron, which should help to reduce intracellular oxidative stress (Ben-Shachar et al. 1991; Jellinger et al. 1992; Good et al. 1992; Ben-Shachar and Youdim 1993; Youdim et al. 1993; Faucheux et al. 2003; Gerlach et al. 2003). The iron-binding protein L-ferritin was also found bound to NM (Tribl et al. 2009). As mentioned above, NM-positive—compared to NM-negative—dopaminergic neurons are more susceptible to PD-induced death but also NM-negative neurons die. NM-negative cells show low iron levels (Hirsch et al. 1994). Investigation of the surviving cells of both types led to the conclusion that the expression of calbindin D28K is positively associated with their survival (Hirsch et al. 1994; Saper 1999). Calbindin D28K controls synaptic calcium dynamics and blocks proapoptotic actions of mutant α-syn/presenilin 1. As a consequence, oxidative stress reduction and preservation of mitochondrial functions follow (Hirsch et al. 1994; Saper 1999).

NM is found in granular vesicles which have alternatively been proposed to be of lysosomal (Tribl et al. 2005; Plum et al. 2016; Wulf et al. 2022a)/auto-lysosomal origin (Zucca et al. 2018). Besides NM pigment, various proteins, iron and other metals, these vesicles can also contain toxins such as MPTP and may have a general function in the isolation of harmful substances. Very recent proteomic analysis showed that NM granules also include proteins typical of stress granules and it was suggested that stress granules might be one of the sources of NM granules (Wulf et al. 2022a, b). In addition, Wulf et al. (2022b) detected α-syn and the protein S100A9 to be enriched in NM granules of Dementia with Lewy Bodies (DLB) cases, while the abundance of several ribosomal proteins was significantly decreased. As S100A9 enhances the formation of α-syn fibrils, this points towards an involvement of NM granules in the pathology of DLB (Mensikova et al. 2022).

While all the above is known about NM, it is not known whether NM essentially is neuroprotective or toxic, or both (Zucca et al. 2008, 2023; Zecca et al. 2015) or why NM-expression in dopaminergic neurons is associated with their increased vulnerability in PD. The increased concentrations of neuronal α-syn, NM cross-linked to α-syn (Fasano et al. 2003) and NM in normal SN neurons may already predispose these neurons to precipitate α-syn around NM-associated lipid under oxidative conditions (Halliday et al. 2005). Therefore, it is not farfetched to assume that the interaction of NM and its iron-induced oxidative stress favors α-syn aggregation leading to LBs, thus causing a toxic cellular environment guilty for the high vulnerability of dopaminergic NM-containing cells of the SNpc.

As mentioned above, the elevated iron levels may induce the production of free radicals, which overwhelms the cellular defense mechanisms ensuing in ferroptosis (Li et al. 2020) or iron-dependent cell destruction in PD (Van Do et al. 2016; Wang et al. 2022). The pathological destruction of the NM-containing dopaminergic neurons leads to a deficit of the iron chelator NM, leading to an elevation of unbound iron and a tide of related cellular damaging events.

NM loss due to the destruction of neurons could have other consequences since it contains about 15% protein in the form of special lysosomal/endosomal (Zecca et al. 2015; Plum et al. 2016) and autophagy proteins (Zucca et al. 2018). This may result in a lysosomal "debt," particularly if there is an underlying genetic fault in the cellular protein homeostasis, which may further heighten the overall burden of misfolded α-syn.

In summary, multiple factors have been published demonstrating a protective role of NM in NM-containing dopaminergic neurons of the SN (Capucciati et al. 2021). However, under certain circumstances, NM binding capacity might be overloaded with toxic compounds, like iron or organic compounds leading to their release when the cytoplasmic fluid composition changes due to metabolic disturbances. The role of granular vesicles in healthy and degenerative phases is under current investigation to enlighten their role as risk factors for neurodegenerative processes, especially the interaction with proteins, incl. α-syn.

## Neuroinflammation in PD

The occurrence of neuroinflammation in the SN of PD was first reported by McGeer et al. (1988) and others (Roca et al. 2011). It may serve as a key player in the pathogenesis or in exacerbating the progression of the illness. However, it is unclear whether it is a cause or consequence of dopaminergic neuronal degeneration. Physiologically, microglia are vital for the protection of the central nervous system via the process of gliosis. Indeed, gliosis is a neuropathological finding in PD post-mortem (Jellinger 2019b). A disturbance in glial cells can unsettle homeostasis and secrete pro- and anti-inflammatory components, resulting in a chronic state of pro-inflammation and deleterious cellular consequences (Ho 2019). Indeed, activated microglia can synthesize and release cytotoxic factors such as cytokines, reactive oxygen/nitrogen species, and prostaglandins.

Furthermore, the cytokines, tumor necrosis factor, and interleukin 1 and 6 (Mogi et al. 1994a, b; Nagatsu et al. 2000 for review) may transform astrocytes into proliferative cells that are summoned to the inflamed brain region (Kettenmann et al. 2011; Fan et al. 2017). Astrocytes (Verkhratsky et al. 2019) play an ambivalent role in physiologically maintaining brain homeostasis. In contrast, they can also adopt a sinister and pathological role by serving as mediators of α-syn neurotoxicity and initiating an inflammatory reaction and defective mitochondrial and proteolytic function (Wang et al. 2021).

A disrupted blood–brain barrier (BBB) may also promote the free passage of the pro-inflammatory mediators from the gut to the brain to induce disruption of cellular mechanisms. The BBB permeability and reduced cerebral blood flow especially in PD with cognitive decline (Derejko et al. 2006; Firbank et al. 2003) involve the neurovascular component managed by endothelial cells that is necessary for the healthy operation of neurons and their pathways. Indeed, BBB leakage has been reported to contribute to cell destruction in the SNpc in animal models of PD (Barcia et al. 2005; Chao et al. 2009), and this establishes the vascular hypothesis for neuronal destruction (Grammas et al. 2011). More importantly, a recent study (Al-Bachari et al. 2017; 2020) has reported a marginal disturbance in the BBB in chief brain regions exhibiting pathology, namely, SN, posterior cortical areas, and white matter. Indeed, a “leaky” BBB is associated with cellular catastrophe, primarily due to the entry of neurotoxins, like iron, and hypoxia, resulting in neurodegeneration (Oestreicher et al. 1994; Faucheux et al. 1999; Montagne et al. 2018). Iron is not only important in the functioning of neurons but also in that of glia (Xu et al. 2018; Song et al. 2018; Reinert et al. 2019). Therefore, it is not farfetched to assume that iron—e.g. taken-up through a leaky BBB at the site of the SN and/or released from both NM and tyrosine hydroxylase (TH) in degenerating DA neurons of the SN—induces pathology resulting in neuroinflammation (Zecca et al. 2008; Zhang et al. 2013; Vila 2019; Salami et al. 2021; Borquez et al. 2022; Ward et al. 2022; Liu et al. 2022).

In summary, evidence supports that neuroinflammation is an early sign of PD. However, the reasons are not well understood and may be based on several confounding pathologies including disturbances in glial functions, like the synthesis of glutathione, cytokines, chemokines, iron-induced oxidative stress, NM released from degenerating neurons, toxins transported through an open BBB, and disturbed immunological reactions. The enigma of neuroinflammation has recently been reviewed by Hirsch and Standaert (2021) and Rolli-Derkinderen et al. (2020).

## Conclusion

Although knowledge from both, basic research and clinical studies has considerably accumulated, the pathology of PD is still an enigma. The reasons for this are manyfold and include lack of information regarding the individual’s multiple genetic risks (which cause specific disturbances in the biochemical/neurobiological metabolic pathways) and—depending on this personal vulnerability for PD—the lack of knowledge regarding the personal triggers that are responsible for the special subtype of this disease. Evidence presented here accumulates to assume (Table 3) that (1) ɑ-syn is a key factor to spread the pathology in a spatiotemporal manner, but (2) seems to be a secondary factor rather than a causal one to trigger sporadic PD, while (3) metabolic initiation factors, like iron, carbon monoxide, viral infections, oxidative stress, reduced antioxidative capacity, mitochondrial dysfunction, proteasomal dysfunction, mitophagy/autophagy and lysosomal dysfunction seem to be of importance to trigger and/or (4) to contribute in a concerted action to PD progression in their multiple combinations.

**Table 3:** Factors in the pathology of sporadic Parkinson’s disease

*Risk factors*: Susceptibility genes, aging, gender, ethnicity, environmental toxins, viral/bacterial infections	
*Single initiation triggers of PD*: Either ɑ-synuclein, iron, oxidative stress, carbon monoxide, viral infections, reduced antioxidative capacity, mitochondrial dysfunction, proteasomal dysfunction, mitophagy/autophagy or lysosomal dysfunction etc	
*Spreading factors*: ɑ-syn, (ß-amyloid, tau-protein in subtypes of PD)	
*Upstream progression factors*: Multiple pathological combination factors like: oxidative stress+-reduced antioxidative capacity+-mitochondrial dysfunction+-proteasomal dysfunction+-mitophagy+-autophagy+-lysosomal dysfunction or any other combination	

Further studies are necessary to enlighten the genetic and epigenetic risk factors leading to sporadic PD. Therapeutic strategies have to be developed to avoid single initiation triggers leading to PD.

## Data Availability

This is a review article based on published literature, no original data was produced, no experiments were made.
